# Supplementary figures and images for: Evolutionary history and classification of Micropia retroelements in Drosophilidae species
Source: PLoS One. 2019 Oct 17;14(10):e0220539. doi: 10.1371/journal.pone.0220539 (PMC6797199; doi:10.1371/journal.pone.0220539)

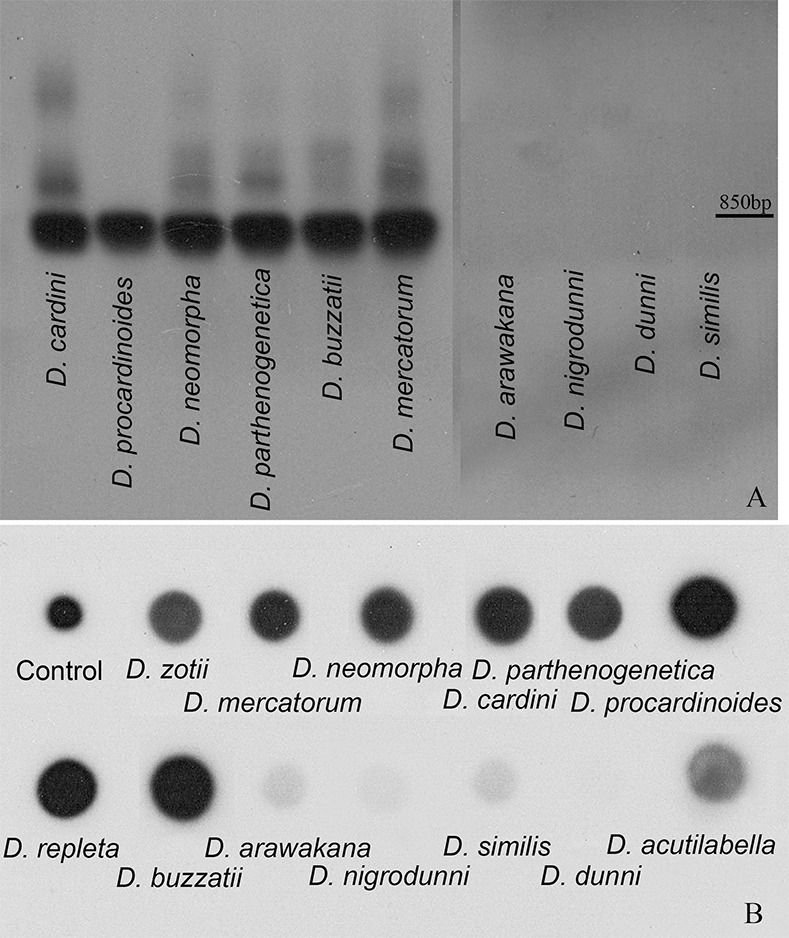

Supplement: S1 Fig — A: PCR-blot results of species from the cardini and repleta groups. B: Dot-blot on genomic DNA confirming the pattern seen on the PCR-blot. In both cases, the probe used was an 812bp PCR fragment from D. hydei dhMiF2 sequence. Control: 5μl (in 10 μl) of the Micropia probe. (TIF) [file pone.0220539.s001.tif]

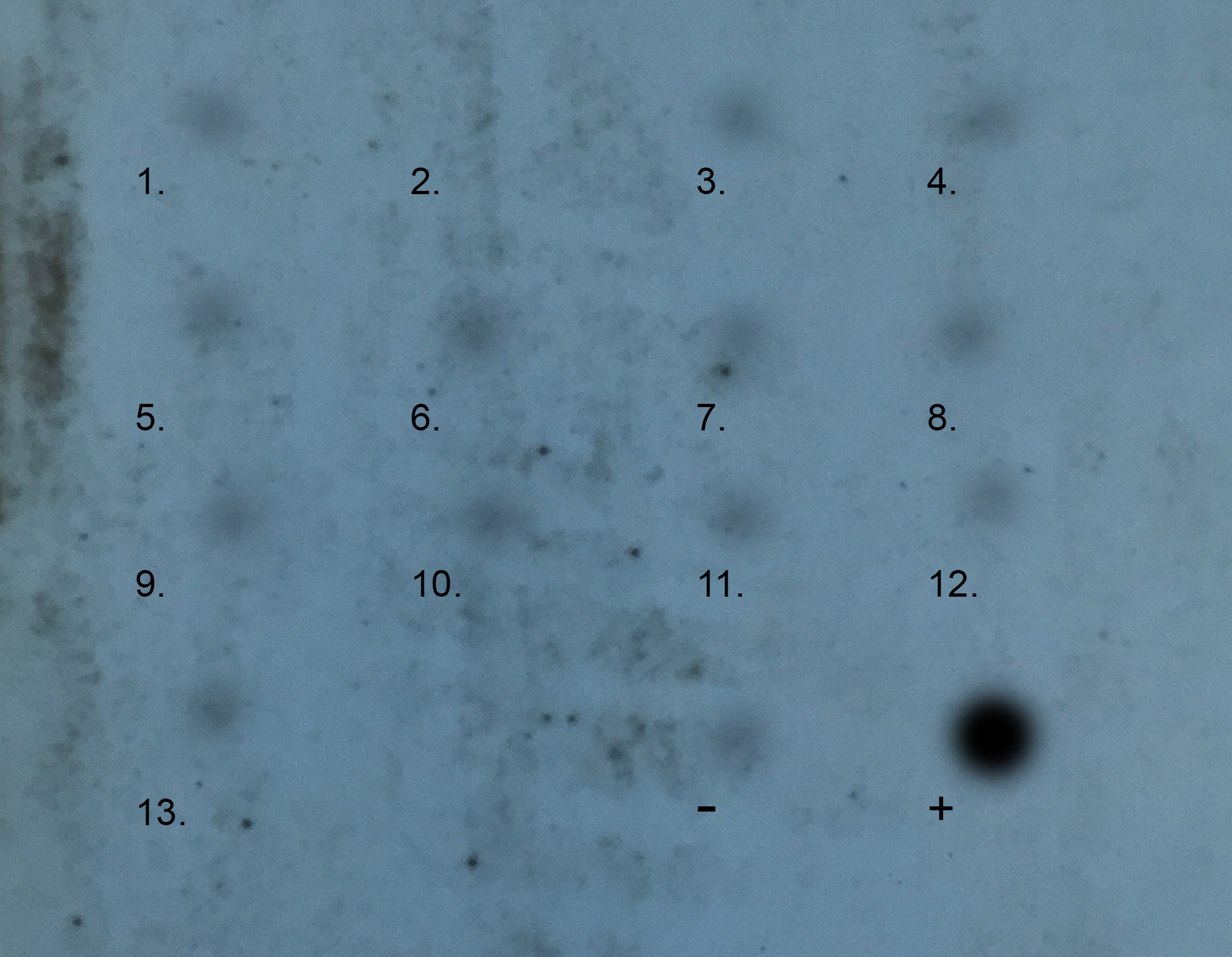

Supplement: S2 Fig — Dot-blot on genomic DNA. The probe used was an 812bp PCR fragment from D. hydei dhMiF2 sequence. 1. D. funnebris; 2. D. griseolineata; 3. D. maculifrons; 4. D. guaru; 5. D. ornatifons; 6. D. immigrans; 7. D. bandeirantorum; 8. D. mediodiffusa; 9. D. mediopictoides; 10. D. mediopunctata; 11. D. paraguayensis; 12. D. paramediostriata; 13. D. tripunctata. +: positive control, 5μl (in 10 μl) of Micropia probe; -: negative control, D. similis DNA. (TIF) [file pone.0220539.s002.tif]
